# Multisite Musculoskeletal Pain and Associated Psychosocial and Occupational Factors in Male Private Security Officers: A Cross-Sectional Study

**DOI:** 10.3390/healthcare14172830

**Published:** 2026-09-03

**Authors:** Ömer Şevgin, Büşra Kesgin, Saniye Koyuncu, Aysu Güngör, Suzan Aydın

**Affiliations:** 1 Department of Physiotherapy and Rehabilitation, Faculty of Health Sciences, Üsküdar University, Istanbul 34662, Türkiye; kesginbusra6@gmail.com (B.K.); ssaniyekoyuncu@gmail.com (S.K.); aysu.gungor.2002@gmail.com (A.G.); 2 Department of Physiotherapy and Rehabilitation, Faculty of Health Sciences, İstanbul Gelişim University, Istanbul 34310, Türkiye; suaydin@gelisim.edu.tr

**Keywords:** occupational health, burnout, sleep quality, shift work, working hours, psychosocial factors, workload, Türkiye

## Abstract

**Background/Objectives**: Private security work combines prolonged standing, extended schedules and shift demands, yet evidence on multisite musculoskeletal pain, burnout and sleep in this group is limited. In this exploratory cross-sectional study, we examined the 12-month prevalence and anatomical distribution of musculoskeletal pain and its associations with burnout, sleep and occupational characteristics. **Methods**: A total of 110 male private security officers in Ümraniye, Istanbul, Türkiye (110/127 approached; participation 86.6%), completed the Nordic Musculoskeletal Questionnaire, Pittsburgh Sleep Quality Index (PSQI) and Maslach Burnout Inventory. Officers reporting pain in ≤2 versus ≥3 regions (multisite pain) were compared, and correlates of multisite pain were examined with binary logistic regression; robustness was assessed with Firth-penalized, count-based, ordinal and re-specified working-hour models. **Results**: Ninety-one participants (82.7%) reported pain in at least one region and 52 (47.3%) in ≥3 regions. Low back pain was most prevalent (54.5%; 95% CI 45.2–63.5), followed by neck and ankle/foot pain (41.8% each). The multisite-pain group had higher emotional exhaustion (14.13 ± 6.26 vs. 10.81 ± 6.77; *p* = 0.009; d = 0.51) and daytime-dysfunction scores (*p* = 0.013), and lower body mass index (*p* = 0.012) and shorter tenure (*p* = 0.026); weekly working hours did not differ significantly in the primary non-parametric comparison. Emotional exhaustion was the only independent correlate in the primary logistic model (OR 1.12, 95% CI 1.02–1.23; *p* = 0.019) and remained associated in Firth-penalized (*p* = 0.029) and covariate-adjusted (*p* = 0.025) analyses, although the association attenuated to borderline significance in count-based, ordinal and categorized working-hour specifications. **Conclusions**: Musculoskeletal symptoms were common and frequently multisite. Emotional exhaustion showed the most consistent—though modest and specification-dependent—association with multisite pain. These exploratory cross-sectional findings should not be interpreted causally.

## 1. Introduction

Musculoskeletal conditions are the leading contributors to disability worldwide, affecting approximately 1.71 billion people, with low back pain representing the single leading cause of disability in many countries [1]. In occupational settings, work-related musculoskeletal disorders (WMSDs) remain an important source of pain, functional limitation, reduced productivity, sickness absence, and healthcare use [2]. Contemporary evidence supports a multifactorial model in which biomechanical exposures interact with psychosocial and organizational hazards rather than acting in isolation [3,4,5]; this is consistent with the biopsychosocial model of chronic pain, in which nociceptive, psychological and social processes jointly shape the pain experience [6].

Private security work combines a distinctive set of physical and organizational demands: prolonged standing and walking, sustained vigilance in static postures, and extended or irregular schedules with limited opportunities for recovery. Biomechanically, prolonged standing and walking are plausible contributors to lower-extremity and spinal symptom burden, although objectively measured ergonomic exposure data for this occupational group remain scarce. Working-time characteristics are also directly relevant: a meta-analysis reported an association between longer working hours and musculoskeletal pain [7], the HUNT study linked shift work with chronic musculoskeletal pain and with the number of painful sites [8], and detailed working-hour characteristics, including shift length and shift variability, have been associated with sickness-absence patterns in airport security personnel [9]. Taken together, the occupational and psychosocial factors so far linked with musculoskeletal pain in security and related protective-service work include long weekly working hours [7], shift work [8], shift length and schedule variability [9], and general psychosocial job hazards such as high demands, low control and low support [3,4,5]; security-specific evidence on burnout and sleep-related factors is summarized below.

Musculoskeletal burden is also increasingly evaluated as a multisite phenomenon. Prospective international evidence indicates that the number of painful anatomical sites may remain relatively stable even when the specific distribution changes over time, suggesting that general susceptibility and systemic or psychosocial factors can contribute alongside region-specific mechanical exposure [10]. The number of painful sites is associated with demographic, lifestyle and health-related factors in a graded fashion, without a single natural threshold [11], and large occupational analyses support classifying multisite pain simply by the number of affected sites [12]. In occupational cohorts, involvement of three or more anatomical sites is among the most commonly applied definitions of multisite pain and has been prospectively associated with sickness absence and restrictions at work [13,14]. Workplace musculoskeletal-health climate has been related to the number of painful sites [15], and longitudinal evidence in younger employees has shown that multisite pain, particularly when accompanied by mental-health burden, is associated with subsequent sickness absence [16]. Thus, counting affected sites may capture clinically and occupationally relevant burden that is not fully represented by analyses of isolated regions.

Burnout is a multidimensional occupational phenomenon characterized principally by emotional exhaustion, depersonalization/cynicism, and reduced professional efficacy or accomplishment [17]. Within the job demands–resources framework, sustained job demands that are not offset by adequate resources deplete energy and give rise to exhaustion, which in turn is linked to health complaints [18]. Sustained occupational demands can affect mental health through several related phenomena—occupational stress, fatigue, monotony, anxiety and depressive symptoms—and these constructs overlap considerably. Among them, burnout was selected as the psychosocial exposure of interest in this study for three reasons: it is by definition work-related, which distinguishes it from generalized anxiety or depressive symptoms; the job demands–resources framework provides an explicit mechanism linking sustained occupational demands to its energetic component and, through it, to health complaints [18]; and a validated Turkish adaptation of an established instrument was available, albeit not formally validated in private security personnel specifically. Within burnout, emotional exhaustion is generally regarded as the core energetic dimension [17] and was therefore specified a priori as the central exposure of interest; other work-related mental-health constructs were not measured, which is considered in the Discussion. Somatic symptoms, including pain, commonly coexist with burnout [19]. Evidence from private security staff has shown substantial levels of burnout and associations with shift work, extended daily schedules, and dissatisfaction with working conditions [20]. More recently, a large study of private security personnel in Türkiye reported that musculoskeletal pain, workload, daily working hours, and sleep-related factors were linked with burnout and psycho-physical distress [21]. In another high-demand occupation, firefighter data suggested that burnout may participate in pathways linking occupational stress and musculoskeletal symptoms [22]. Law-enforcement research has likewise demonstrated a high 12-month prevalence of musculoskeletal complaints, particularly in the low back, shoulder, and neck [23].

Despite these converging findings, evidence integrating the anatomical distribution of musculoskeletal pain with burnout, sleep quality, and occupational characteristics specifically among private security officers remains limited: existing studies in this occupational group have examined burnout or psychological distress as the outcome [20,21], whereas, to our knowledge, no previous study has taken the anatomical distribution and multisite burden of musculoskeletal pain as the primary outcome and tested its psychosocial and occupational correlates in private security personnel. The general objective of this exploratory study was therefore to characterize the 12-month burden of musculoskeletal pain among male private security officers and its psychosocial and occupational correlates. This objective was specified in three hypotheses: (H1) musculoskeletal pain would be common and would frequently involve multiple (≥3) anatomical regions; (H2) officers with multisite pain would show higher burnout subscale scores (particularly emotional exhaustion), poorer sleep quality (global PSQI and component scores), and heavier working-time exposure (longer weekly and daily working hours, more monthly working days, and longer tenure in private security); and (H3) emotional exhaustion would remain independently associated with multisite pain after mutual adjustment for burnout, sleep, and occupational variables. In view of the convenience sampling and the absence of an a priori sample-size calculation, all association analyses were regarded as exploratory.

## 2. Materials and Methods

### 2.1. Study Design, Setting, and Participants

This observational cross-sectional study was conducted among private security officers working in the Ümraniye district of Istanbul, Türkiye. Participating workplaces comprised shopping malls, hospitals and other private commercial premises, so the sample covers a range of security roles rather than a single institutional setting. Institutional permission was obtained from each participating workplace before recruitment. Data were collected between May and December 2025 through the Üsküdar University Physiotherapy and Rehabilitation Application and Research Center (ÜSFİZYOTEM). Officers were approached through written study announcements and on-site briefings. Questionnaires were administered through structured face-to-face interviews conducted individually at the workplace by trained members of the research team, who read the items aloud and recorded the participants’ responses; this approach standardized the administration procedure and minimized missing or misunderstood items. Before data collection, the interviewers were trained by the supervising academic author (Ö.Ş.) in dedicated sessions covering the content and standardized administration of the study instruments, a neutral, non-suggestive interviewing style, and the confidentiality procedures; the initial interviews were then conducted under supervision as a pilot to verify consistent administration before independent data collection began, and periodic checks of completed forms were performed throughout the study period. The study is reported in accordance with the Strengthening the Reporting of Observational Studies in Epidemiology (STROBE) statement for cross-sectional studies, and the completed checklist is provided as Supplementary Materials (Table S1); the participant flow is reported in Section 3.1 and Figure S1.

No a priori sample-size calculation was performed. Recruitment was based on a convenience sample of officers accessible at the participating workplaces during the study period, so the achieved sample size was determined by availability rather than by a target power. The study should therefore be regarded as exploratory with respect to statistical power.

Eligible participants were men currently employed as private security officers at one of the participating workplaces in the Ümraniye district who had completed at least one year of active employment in private security and were able to read Turkish. Officers holding an additional concurrent job were excluded. No further age restriction was applied; participants’ ages ranged from 21 to 56 years. Participants received verbal and written information about the study and provided written informed consent before data collection. Participation was voluntary, responses were anonymous, and participants were informed that their decision to participate or decline would have no effect on their employment.

### 2.2. Measures

#### 2.2.1. Sociodemographic and Occupational Characteristics

A structured form was used to record age, sex, body mass index (BMI), marital status, educational level, smoking status, duration of employment in private security, daily working hours, weekly working hours, and the number of working days per month. Detailed shift-schedule characteristics—shift type (day/night/rotating), shift sequence, rest intervals between consecutive shifts, and the number of night shifts—were not recorded; working-time exposure is therefore represented by daily and weekly working hours and monthly working days, which is addressed as a limitation.

#### 2.2.2. Musculoskeletal Symptoms

Musculoskeletal symptoms were assessed using the Nordic Musculoskeletal Questionnaire (NMQ), an established occupational-health screening instrument [24]. The Turkish adaptation has demonstrated satisfactory psychometric properties [25]. For each of the nine anatomical regions (neck, shoulders, upper back, elbows, wrists/hands, low back, hips/thighs, knees, and ankles/feet), participants reported whether they had experienced pain, ache, or discomfort during the preceding 12 months. The present analyses were based on the 12-month symptom responses. The resulting mismatch between the 12-month pain recall window and the shorter reference periods of the sleep and burnout measures is addressed in the limitations.

#### 2.2.3. Sleep Quality

Sleep quality was assessed with the Pittsburgh Sleep Quality Index (PSQI) [26], using the Turkish version validated by Ağargün et al. [27]. The PSQI evaluates sleep during the previous month across seven components: subjective sleep quality, sleep latency, sleep duration, habitual sleep efficiency, sleep disturbances, use of sleep medication, and daytime dysfunction. Component scores range from 0 to 3 and sum to a global score of 0–21, with higher scores indicating poorer sleep quality. A global score > 5 is conventionally used to indicate poor sleep quality. Because item-level responses were not retained in the analysis dataset, internal-consistency coefficients were not recomputed in the present sample, and reliability rests on the published Turkish validation [27].

#### 2.2.4. Burnout

Occupational burnout was assessed using the Maslach Burnout Inventory (MBI), originally developed by Maslach and Jackson [28] and adapted for Turkish use by Ergin [29]. The administered version comprised 22 items rated from 0 (never) to 4 (always), organized into emotional exhaustion (9 items), depersonalization (5 items), and personal accomplishment (8 items). The Turkish adaptation uses a 5-point (0–4) frequency scale rather than the original 7-point (0–6) scale, so absolute subscale scores are not directly comparable with international reports. The three subscale scores were analyzed separately as the burnout measures, using raw (non-reversed) item scores. Higher emotional-exhaustion and depersonalization scores indicate greater burnout, whereas the personal-accomplishment subscale is scored in the opposite direction, so that lower scores indicate reduced perceived accomplishment and therefore greater burnout on this dimension. Consistent with the recommendations of the instrument developers, no composite total burnout score was computed. Because the MBI subscales represent distinct constructs, a reliability coefficient computed across the full instrument would not be informative; as only subscale totals were retained in the analysis dataset, subscale-specific internal-consistency coefficients could not be computed in the present sample, and reliability rests on the published Turkish adaptation [29]. This, and the fact that the measurement properties of the MBI have not been formally established in private security personnel, are addressed as limitations.

### 2.3. Definition of Multisite Musculoskeletal Pain

For the primary analytical outcome, the number of anatomical regions with 12-month musculoskeletal pain was counted (range 0–9). Participants were categorized as having pain in ≤2 anatomical regions or multisite musculoskeletal pain involving ≥3 regions, with the latter coded as the event in the binary logistic regression analysis. There is no universally agreed threshold for defining multisite pain, and the number of painful sites relates to health outcomes in a graded fashion [11,12]. Involvement of three or more sites is nevertheless among the most widely used definitions in occupational studies and identifies a pain burden that has been prospectively associated with sickness absence and restrictions at work [13,14]; in the present sample, this threshold also corresponds to a burden above the mean number of symptomatic regions (2.75). To reduce the dependence of the findings on this cut-off, the number of painful regions was additionally analyzed as a count and as an ordinal outcome (Section 2.4).

### 2.4. Statistical Analysis

Analyses were performed using IBM SPSS Statistics for Windows, version 22.0 (IBM Corp., Armonk, NY, USA), and were independently reproduced in Python, version 3.11 (Python Software Foundation, Wilmington, DE, USA), using the pandas (version 3.0.2), statsmodels (version 0.14.6), and SciPy (version 1.17.1) libraries. All figures were generated directly from the study dataset using the Matplotlib library, version 3.10.9 (Matplotlib Development Team). During the preparation of this revision, the full dataset was re-audited against the original paper questionnaires: data-entry errors (including mixed measurement units in the height field and isolated out-of-range codes) were corrected, scale totals were recomputed from their components, and all analyses were rerun on the audited dataset; the values reported here therefore supersede those in the originally submitted version. Data quality control comprised range checks for every variable, cross-checks of scale totals against component scores, and logical-consistency checks (e.g., employment duration versus age); four records with implausible or internally inconsistent occupational values were retained in the primary analysis and examined in a sensitivity analysis excluding them. Continuous variables are summarized as mean ± standard deviation (SD) and categorical variables as number and percentage; 95% confidence intervals (CIs) for prevalence estimates were calculated using the Wilson score method. For two-group comparisons, the primary test for each variable was designated according to measurement level and distribution: the independent-samples *t* test was primary where the Shapiro–Wilk test indicated approximate normality in both groups, and the Mann–Whitney U test was primary for ordinal variables (the PSQI component scores) and for variables departing from normality. The non-primary test is reported alongside for transparency, and inference is based on the primary test throughout. Cohen’s d is reported as a standardized effect size. Region-specific and subscale comparisons were exploratory and are reported with Benjamini–Hochberg false-discovery-rate (FDR)-adjusted *p* values in addition to unadjusted values.

The binary logistic regression model examined factors associated with pain in ≥3 regions. The number of explanatory variables was constrained by the number of events (*n* = 52), corresponding to approximately seven events per variable, and the seven predictors were specified a priori on conceptual grounds: the two burnout dimensions central to occupational strain (emotional exhaustion and depersonalization), the three PSQI components most directly related to perceived sleep quality, pharmacological sleep aid, and daytime recovery (subjective sleep quality, sleep-medication use, and daytime dysfunction), and the two occupational exposures with the strongest a priori link to cumulative loading (years of employment in private security and weekly working hours). Because age and BMI are established correlates of musculoskeletal pain, a pre-specified sensitivity model additionally adjusted for these two covariates. The linearity of continuous predictors in the logit was examined with the Box–Tidwell procedure. Model robustness was further examined with (i) Firth penalized logistic regression; (ii) a model in which weekly working hours—which took only six distinct values—were categorized (≤45, 46–54, and 60 h/week); (iii) negative binomial regression of the number of painful regions (0–9), chosen over Poisson regression because of overdispersion; (iv) ordinal logistic regression across pain-burden categories (0, 1–2, 3–4, and ≥5 regions); (v) modeling emotional exhaustion in quartiles, with bootstrap percentile 95% CIs (5000 resamples) computed for the quartile odds ratios in view of the small number of participants per quartile; and (vi) re-estimation after excluding the four records flagged in the data-quality checks. Multicollinearity was assessed with variance inflation factors (VIFs). Odds ratios (ORs) with 95% CIs were reported; overall model significance was evaluated using the Omnibus test and calibration using the Hosmer–Lemeshow test, and Nagelkerke R^2^ and classification accuracy were also reported. Because no a priori sample-size calculation was performed, the precision and magnitude of the findings are conveyed by the reported effect sizes and 95% confidence intervals rather than by post hoc power statistics. All tests were two-sided, exact *p* values are reported, *p* < 0.05 was considered statistically significant, and all association analyses are interpreted as exploratory.

### 2.5. Ethical Considerations

The study was approved by the Üsküdar University Non-Interventional (Non-Clinical) Research Ethics Committee (Meeting No. 04, 29 April 2025; decision no. 61351342/020-1109) and was conducted in accordance with the Declaration of Helsinki. Written informed consent for participation was obtained from all participants before data collection.

## 3. Results

### 3.1. Participant Characteristics

Of the 127 officers approached, 9 did not meet the eligibility criteria and 4 declined to participate; of the remaining 114 who completed the assessment, 4 were excluded because of incomplete questionnaires. The final analytic sample therefore comprised 110 male private security officers, corresponding to a participation rate of 86.6% of all officers approached and 93.2% of those eligible (Figure S1). Mean age was 36.94 ± 9.02 years (range 21–56) and mean body mass index was 24.53 ± 3.26 kg/m^2^ (range 19.29–33.95). Participants had worked in private security for 11.02 ± 8.45 years, worked 10.64 ± 1.84 h/day and 49.40 ± 9.08 h/week, and worked 20.82 ± 5.52 days per month. Most were married (56.4%) and had completed high school (64.5%); 33.6% were current smokers (Table 1).

### 3.2. Twelve-Month Distribution and Burden of Musculoskeletal Pain

**Figure 1:**
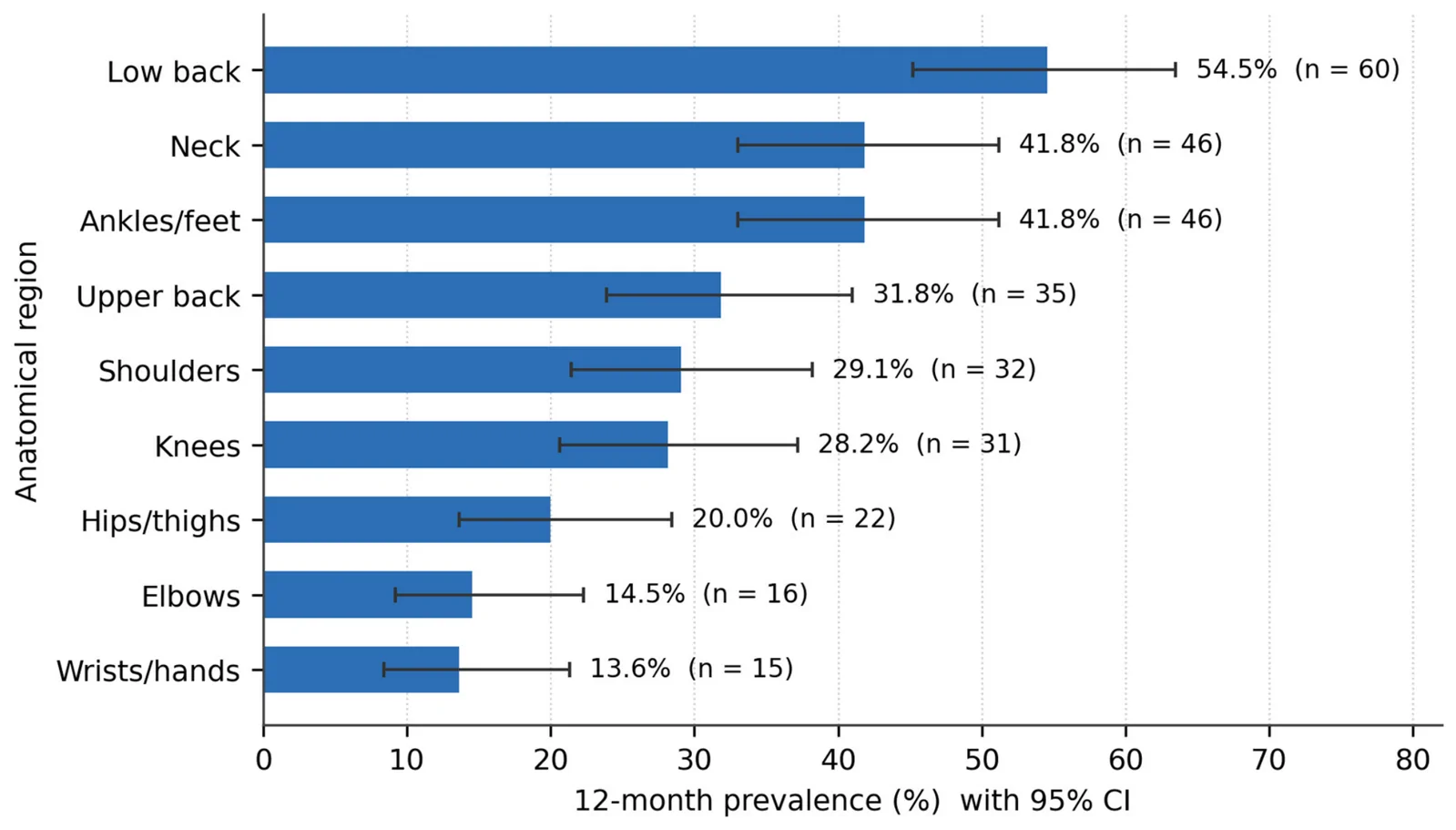
Twelve-month prevalence of musculoskeletal pain by anatomical region among private security officers (*n* = 110). Error bars represent 95% confidence intervals calculated using the Wilson score method.

**Figure 2:**
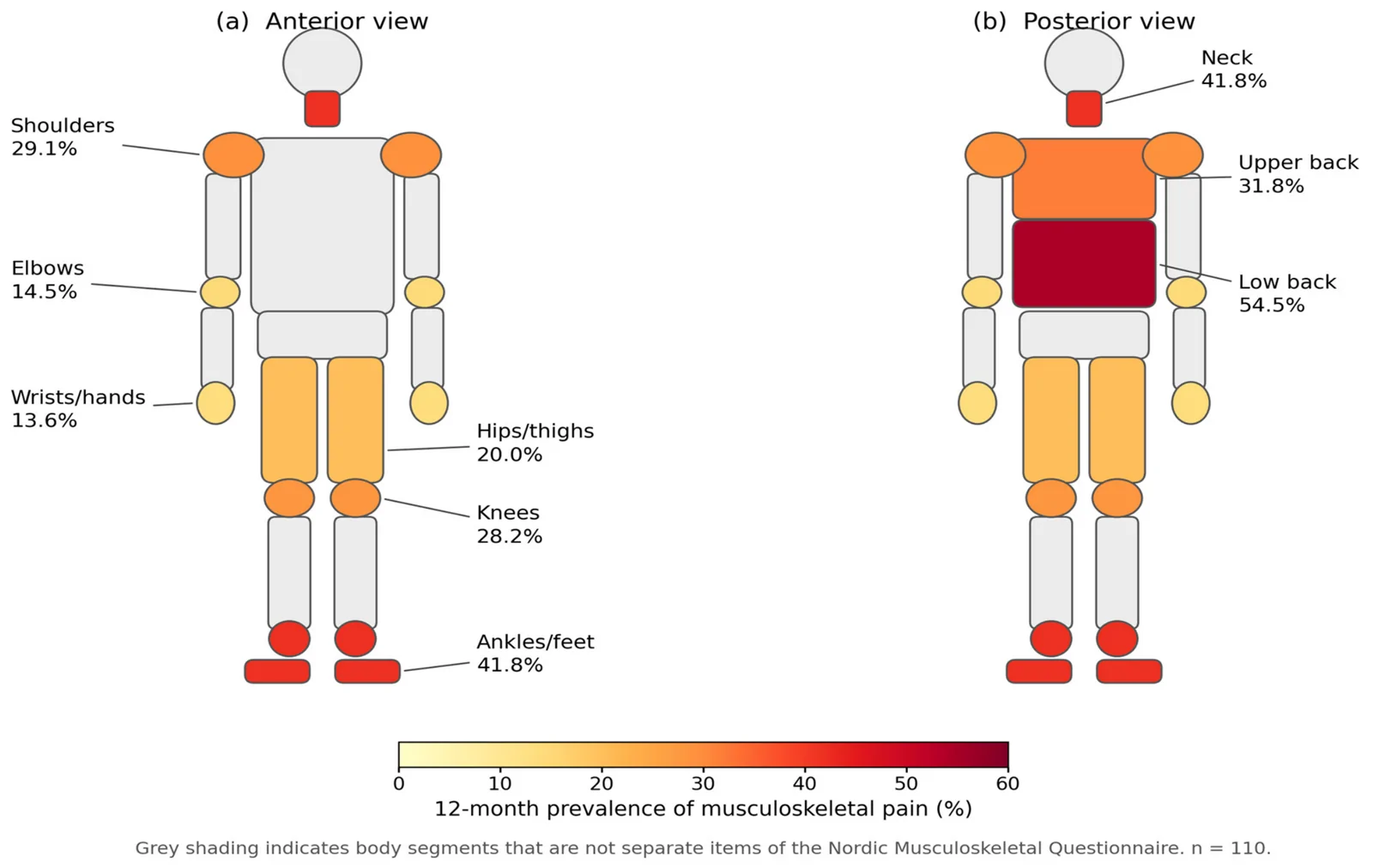
Anatomical symptom map of 12-month musculoskeletal pain prevalence (*n* = 110). Color intensity is proportional to the prevalence of self-reported pain, ache, or discomfort in each Nordic Musculoskeletal Questionnaire region. The greatest symptom burden was observed in the low back, neck, and ankles/feet.

Low back pain was the most frequently reported 12-month musculoskeletal symptom, affecting 60 participants (54.5%; 95% CI 45.2–63.5). Neck and ankle/foot pain were each reported by 46 participants (41.8%; 95% CI 33.0–51.2). Upper-back symptoms were reported by 35 (31.8%), shoulder pain by 32 (29.1%), knee pain by 31 (28.2%), hip/thigh pain by 22 (20.0%), elbow pain by 16 (14.5%), and wrist/hand pain by 15 (13.6%) (Table 2). Ninety-one participants (82.7%; 95% CI 74.6–88.7) reported pain in at least one anatomical region during the preceding 12 months, whereas 19 (17.3%) reported no symptoms in any region. The mean number of symptomatic regions was 2.75; 19 participants (17.3%) reported no symptomatic region, 39 (35.5%) one or two regions, 27 (24.5%) three or four regions, and 25 (22.7%) five or more regions. Fifty-eight participants (52.7%; 95% CI 43.5–61.8) reported pain in ≤2 regions, whereas 52 (47.3%; 95% CI 38.2–56.5) reported pain in ≥3 regions. The regional prevalence and its distribution on an anatomical symptom map are shown in Figure 1 and Figure 2.

### 3.3. Burnout, Sleep, and Occupational Characteristics According to Multisite Pain

Under the prespecified testing strategy, participants with multisite pain had higher emotional-exhaustion scores than those with pain in ≤2 regions (14.13 ± 6.26 vs. 10.81 ± 6.77; mean difference 3.32, 95% CI 0.86 to 5.78; *t* test *p* = 0.009; d = 0.51; Mann–Whitney *p* = 0.014) and higher PSQI daytime-dysfunction scores (0.63 ± 0.71 vs. 0.40 ± 0.79; Mann–Whitney *p* = 0.013). Body mass index (23.83 ± 3.35 vs. 25.15 ± 3.07 kg/m^2^; Mann–Whitney *p* = 0.012; d = −0.41) and years of employment in private security (9.40 ± 8.25 vs. 12.47 ± 8.44 years; Mann–Whitney *p* = 0.026; d = −0.37) were lower in the multisite-pain group. Weekly working hours were higher in the multisite-pain group in the parametric comparison (51.52 ± 8.74 vs. 47.50 ± 9.04; *t* test *p* = 0.020; d = 0.45), but this variable departed from normality and its primary non-parametric comparison was not statistically significant (Mann–Whitney *p* = 0.071); weekly working hours are therefore not interpreted as showing a significant group difference. Depersonalization (*p* = 0.202), personal accomplishment (*p* = 0.376; because this subscale is reverse-scored, the slightly higher value in the multisite-pain group corresponds to marginally greater perceived accomplishment rather than greater burnout), the global PSQI score (3.92 ± 2.90 vs. 3.21 ± 2.81; *p* = 0.159), age (35.23 ± 8.91 vs. 38.47 ± 8.92; *t* test *p* = 0.060), daily working hours (*p* = 0.147) and monthly working days (*p* = 0.670) did not differ significantly between the groups (Table 3). Overall, 26 participants (23.6%) exceeded the conventional PSQI cut-off of >5 for poor sleep quality, and the sample mean of 3.55 lay below this threshold.

Exploratory region-specific comparisons (9 regions × 11 variables = 99 tests) identified nine differences at unadjusted *p* < 0.05 under the primary tests, including higher emotional exhaustion among participants with ankle/foot pain (*p* = 0.016) and with shoulder pain (*p* = 0.028), and lower age (*p* = 0.018), body mass index (*p* = 0.001) and tenure (*p* = 0.002) among those with ankle/foot pain. After Benjamini–Hochberg correction, none of the 99 comparisons remained statistically significant (lowest adjusted *p* = 0.082). These site-specific findings are therefore hypothesis-generating only; the complete set of comparisons with parametric, non-parametric and adjusted *p* values is provided in Supplementary Table S2. In additional exploratory subgroup analyses, neither the number of painful regions nor the global PSQI score differed across marital status, smoking status, or educational level (all FDR-adjusted *p* ≥ 0.92; Table S3).

### 3.4. Multivariable Analysis of Multisite Musculoskeletal Pain

**Figure 3:**
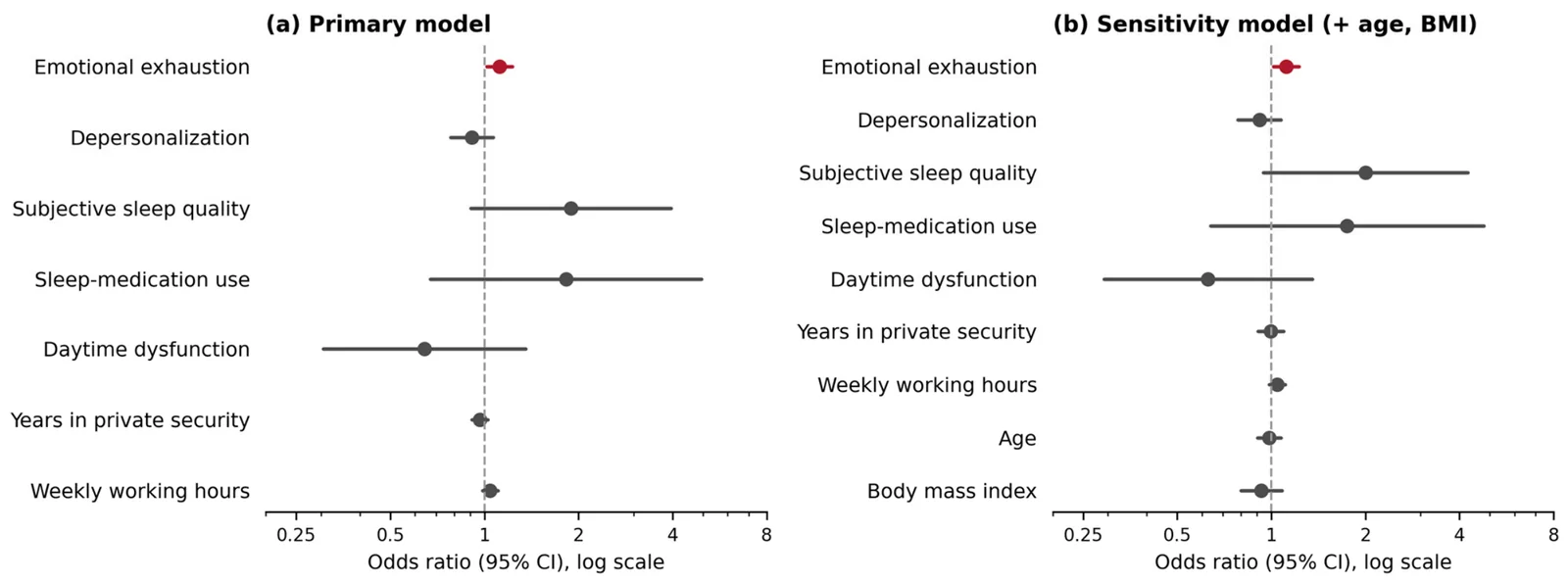
Multivariable logistic-regression estimates for multisite musculoskeletal pain (pain in ≥3 anatomical regions; 52 events). (**a**) Primary model; (**b**) sensitivity model additionally adjusted for age and body mass index. Points are odds ratios and horizontal lines are 95% confidence intervals on a logarithmic scale; the dashed line marks the null value of 1. Estimates significant at *p* < 0.05 are shown in red.

The primary binary logistic regression model was statistically significant (Omnibus χ^2^(7) = 17.498, *p* = 0.014), with Nagelkerke R^2^ = 0.196 and an overall classification accuracy of 69.1%, compared with 52.7% in the intercept-only model. Emotional exhaustion was the only statistically significant independent correlate: each one-point increase in emotional-exhaustion score was associated with 12% higher odds of reporting pain in ≥3 regions (OR 1.12, 95% CI 1.02–1.23; *p* = 0.019). Subjective sleep quality, sleep-medication use, daytime dysfunction, depersonalization, years in private security, and weekly working hours were not independently associated with the outcome (Table 4 and Figure 3a). The Hosmer–Lemeshow test indicated imperfect calibration for this model (χ^2^(8) = 17.494, *p* = 0.025); given the small number of events, this statistic should be interpreted with caution, and calibration was adequate in the sensitivity and robustness models described below.

In the pre-specified sensitivity analysis additionally adjusted for age and body mass index, the model remained significant (Omnibus χ^2^(9) = 18.720, *p* = 0.028; Nagelkerke R^2^ = 0.209; classification accuracy 65.5%; Hosmer–Lemeshow χ^2^(8) = 7.506, *p* = 0.483) and emotional exhaustion retained its association with multisite pain (OR 1.11, 95% CI 1.01–1.22; *p* = 0.025). Neither age (OR 0.98, 95% CI 0.90–1.07; *p* = 0.698) nor body mass index (OR 0.93, 95% CI 0.80–1.08; *p* = 0.337) was independently associated with the outcome, and no other variable reached significance (Table 5 and Figure 3b). On the audited dataset, the Box–Tidwell procedure provided no evidence against linearity in the logit for any continuous predictor (all interaction *p* ≥ 0.105; weekly working hours *p* = 0.745); the categorized working-hours model described in Section 3.5 was nevertheless retained as a robustness analysis because of the small number of distinct values of this variable.

### 3.5. Robustness and Sensitivity Analyses

The direction of the emotional-exhaustion association was consistent across all specifications, whereas its statistical significance was specification-dependent. The association persisted in Firth penalized regression (OR 1.11, 95% CI 1.01–1.21; *p* = 0.029) and after excluding the four records flagged in the data-quality checks (*n* = 106; OR 1.12; *p* = 0.018). When weekly working hours were modeled categorically (≤45, 46–54, and 60 h/week), overall model fit and calibration improved (Omnibus χ^2^(8) = 21.638, *p* = 0.006; Nagelkerke R^2^ = 0.238; Hosmer–Lemeshow *p* = 0.496), and the emotional-exhaustion estimate was essentially unchanged in magnitude but attenuated to borderline significance (OR 1.09, 95% CI 0.99–1.20; *p* = 0.072); no working-hours category was independently significant in this fully adjusted model. In negative binomial regression of the number of painful regions, the emotional-exhaustion estimate was again positive but not statistically significant (incidence rate ratio 1.03, 95% CI 0.99–1.06; *p* = 0.122), as was the case in ordinal logistic regression across pain-burden categories (OR 1.07, 95% CI 0.99–1.15; *p* = 0.078).

Modeling emotional exhaustion in quartiles suggested a threshold above the lowest quartile rather than a strictly graded dose–response pattern: relative to the lowest quartile, the odds of multisite pain were elevated in the second (OR 4.09, 95% CI 1.16–14.37; *p* = 0.028), third (OR 3.39, 95% CI 0.86–13.40) and highest (OR 4.04, 95% CI 0.82–19.90) quartiles. These estimates rest on only 26–29 participants per quartile and are correspondingly imprecise: bootstrap percentile 95% CIs (5000 resamples) were 1.02–27.12, 0.74–23.33 and 0.62–40.84, respectively, confirming that the intervals for the third and highest quartiles clearly include the null and that even the second-quartile interval approaches it. The quartile analysis is therefore reported only as a coarse check on the shape of the association; it does not by itself establish elevated odds in any individual quartile, and the suggested threshold pattern should not be over-interpreted. Variance inflation factors indicated no problematic multicollinearity (all VIFs ≤ 2.07). Taken together, these analyses indicate a consistently positive but modest association between emotional exhaustion and multisite pain whose statistical significance depends on model specification; given approximately seven events per variable and the exploratory design, this association should be regarded as suggestive rather than established.

## 4. Discussion

This study examined the 12-month anatomical distribution of musculoskeletal pain among male private security officers while simultaneously considering burnout, sleep, and occupational characteristics. Three findings are particularly important. First, musculoskeletal pain was common and frequently multisite: 82.7% of participants reported pain in at least one region and 47.3% in at least three, with a mean of 2.75 symptomatic regions per officer. Second, low back pain was the most prevalent regional complaint, followed by neck and ankle/foot pain. Third, emotional exhaustion showed the most consistent association with multisite pain among the variables studied: it was the only independent correlate in the primary logistic model and remained associated in Firth-penalized and covariate-adjusted analyses, although the association attenuated to borderline significance in count-based, ordinal and categorized working-hour specifications. With respect to the prespecified hypotheses, H1 was supported; H2 was only partially supported—emotional exhaustion and daytime dysfunction were higher in the multisite-pain group, but global sleep quality, daily working hours and monthly working days did not differ, the difference in weekly working hours was not confirmed by the primary non-parametric test, and tenure showed an association opposite in direction to that hypothesized; and H3 was supported in the primary and penalized models but was specification-dependent (Section 3.5). Because biomechanical and ergonomic exposures were not measured, these findings cannot be used to compare psychosocial with biomechanical explanations of musculoskeletal pain; rather, they are consistent with a multidimensional occupational-health perspective in which psychosocial factors operate alongside—not instead of—physical and organizational exposures [2,3,4,5].

The predominance of low back and neck complaints is consistent with findings in occupational groups that share prolonged standing, equipment use, vigilance, or law-enforcement-related demands. In English law-enforcement officers, Kasem et al. reported 12-month complaint prevalences of 59.1% for the lower back, 48.4% for the shoulders, and 42.5% for the neck [23]. The low-back and neck estimates in the present study (54.5% and 41.8%, respectively) are therefore similar in magnitude despite differences in role and setting. Shoulder complaints, by contrast, were considerably less frequent in our sample (29.1% vs. 48.4%), which may reflect differences in equipment load: law-enforcement officers routinely carry duty belts, body armor and communication equipment that impose sustained shoulder loading, whereas private security officers in commercial premises typically do not. The relatively high ankle/foot prevalence (41.8%) is additionally noteworthy. Private security work often requires sustained standing and walking, but the present study did not objectively measure standing duration, step counts, footwear, or lower-extremity loading; consequently, biomechanical explanations for this pattern remain hypotheses for future research rather than demonstrated mechanisms. A comparable lower-extremity and low-back symptom profile has been described in other occupations characterized by prolonged standing, such as supermarket cashiers, in whom combined ergonomic training and exercise reduced musculoskeletal pain and ergonomic risk [30].

The high frequency of multisite pain may be more informative than region-by-region prevalence alone. Ntani et al. found that the number of painful sites showed substantial stability over time across diverse occupational groups even when specific anatomical sites changed [10], and the number of affected sites relates to demographic and health-related factors in a graded fashion [11,12]. This supports the concept that multisite pain can reflect broader susceptibility in addition to localized mechanical exposure. Organizational context may also matter: musculoskeletal-health climate has been associated with the number of painful sites among workers [15]. Furthermore, multisite pain has been prospectively associated with sickness absence and restrictions at work in occupational cohorts [13,14], and register-based longitudinal evidence has linked multisite pain, particularly when combined with mental-health burden, with subsequent sickness absence [16]. In the current sample, the finding that almost one in two officers reported pain in ≥3 regions therefore suggests a clinically and occupationally meaningful burden rather than simply a collection of isolated regional complaints.

Emotional exhaustion was the clearest psychosocial correlate of multisite pain. The multisite-pain group had significantly higher emotional-exhaustion scores, with a moderate standardized effect size (d = 0.51), and this dimension remained associated with the outcome in the primary model (OR 1.12, 95% CI 1.02–1.23), in the Firth-penalized model (OR 1.11) and after additional adjustment for age and body mass index (OR 1.11, 95% CI 1.01–1.22). At the same time, the estimate attenuated to borderline significance in the count-based, ordinal and categorized working-hours specifications (Section 3.5), and the explanatory capacity of all models was modest (Nagelkerke R^2^ ≈ 0.20); we therefore interpret this association cautiously and in proportion to these limits throughout the manuscript. Burnout is inherently multidimensional, and emotional exhaustion is generally considered its core energetic component [17]. Somatic symptoms, including pain, have also been shown to cluster with burnout in population-based data [19]. In private security staff, Veljković et al. identified shift work, extended daily work, and dissatisfaction with working conditions as important correlates of burnout [20]. Bener et al. [21] further demonstrated in private security personnel in Türkiye that burnout, emotional exhaustion, sleep problems, working hours, workload, and musculoskeletal pain are interrelated within this occupational group. A comparable clustering has been reported in Turkish physiotherapists, among whom burnout, occupational satisfaction and musculoskeletal pain were interrelated within the same workforce [31]. Our analysis addresses the relationship from the complementary perspective of multisite pain as the outcome; the direction of the association (approximately 9–12% higher odds of multisite pain per emotional-exhaustion point across specifications) is compatible with these reports, although differences in instruments, scales and outcome definitions preclude direct numerical comparison of effect sizes across studies. It should also be emphasized that these findings concern the emotional-exhaustion dimension rather than the full burnout syndrome. Exhaustion is typically the first-developing and most prevalent component of burnout, particularly in service-sector occupations, and it overlaps conceptually with occupational stress and fatigue; in the multidimensional model, burnout as a syndrome is present only when exhaustion co-occurs with depersonalization and reduced personal accomplishment [17]. Because neither depersonalization nor personal accomplishment differed between the pain groups or contributed to the models, the present results should be read as an association between exhaustion—a relatively non-specific indicator of depleted energetic resources—and multisite pain, and not as evidence that the full burnout syndrome characterizes officers with multisite pain. Related constructs that were not measured here, such as occupational stress, fatigue or monotony, may capture the same underlying process and deserve direct comparison with exhaustion in future studies.

Evidence from other physically and psychologically demanding occupations supports the plausibility of this association. Khoshakhlagh et al. reported relationships among occupational stress, burnout, and musculoskeletal disorders in firefighters, with burnout contributing to modeled pathways between occupational stress and musculoskeletal symptoms [22]. Nevertheless, the cross-sectional design of our study does not establish directionality. Emotional exhaustion may increase pain sensitivity or reduce recovery, persistent pain may contribute to exhaustion, or both may arise from common upstream exposures such as workload, recovery constraints, psychosocial stress, or work organization. Accordingly, the observed OR should be interpreted as an association in odds rather than a causal risk estimate.

Weekly working hours were higher among officers with multisite pain in the parametric comparison, but this difference was not confirmed by the primary non-parametric test (*p* = 0.071), and weekly working hours were not independently associated with multisite pain in any multivariable specification. Weekly working hours took only six distinct values in this sample, which limits the resolution of this variable; in the categorized analysis, officers working 60 h/week had elevated odds relative to those working ≤45 h/week in a parsimonious model (OR 2.77, 95% CI 1.13–6.83), but this estimate was not significant in the fully adjusted model and should be regarded as hypothesis-generating. These observations are directionally consistent with meta-analytic evidence linking longer working hours to musculoskeletal pain [7], with large occupational data showing associations between shift work and both chronic musculoskeletal pain and the number of painful sites [8], and with security-specific research associating working-hour characteristics with sickness absence [9]; however, no independent association was demonstrated in the present data. Monthly working days did not differ between the pain groups (20.50 ± 6.72 vs. 21.17 ± 3.79; *p* = 0.670). Detailed shift characteristics—shift type, shift sequence, the rest interval between consecutive shifts, and night-shift frequency—were not recorded and may be more informative markers of circadian and recovery burden than aggregate working time.

Two unexpected patterns deserve comment. Officers with multisite pain had a lower body mass index than those with pain in ≤2 regions (23.83 ± 3.35 vs. 25.15 ± 3.07 kg/m^2^; *p* = 0.012; d = −0.41) and fewer years of employment in private security (9.40 ± 8.25 vs. 12.47 ± 8.44 years; *p* = 0.026; d = −0.37). Both gradients run opposite to what a simple cumulative-exposure or mechanical-loading model would predict, and neither variable was independently associated with the outcome in the adjusted models. The most plausible explanation is a healthy-worker survivor effect: officers who develop disabling musculoskeletal symptoms may transfer to lighter duties or leave the sector, so that those with the longest tenure represent a selected, more resilient group. It should be emphasized, however, that this explanation was not directly assessed in this cross-sectional study and remains a hypothesis. An alternative interpretation is that a lower body mass index within this largely normal-weight range may partly reflect lower muscle mass or physical conditioning rather than lower mechanical loading; because body composition was not measured, these explanations cannot be distinguished. These findings should therefore not be read as indicating that longer service or higher body mass protects against musculoskeletal pain; rather, the prevalence reported here may underestimate the true burden in the wider private-security workforce.

Sleep findings were more nuanced. The global PSQI score did not differ significantly between the two groups (3.92 ± 2.90 vs. 3.21 ± 2.81; *p* = 0.159) and no sleep variable was a significant independent correlate in the final models, although daytime dysfunction was higher in the multisite-pain group in the primary univariate comparison (*p* = 0.013). It is relevant that overall sleep quality in this sample was comparatively good (mean global PSQI 3.55, with 26 participants (23.6%) exceeding the conventional cut-off of >5); the resulting restriction of range may have limited the ability to detect an association with multisite pain and represents a plausible floor effect. Broader evidence indicates a bidirectional relationship between sleep problems and chronic musculoskeletal pain [32,33], and a systematic review focused on workers similarly identified links between sleep disturbance and musculoskeletal disorders [34]. In emergency workers, sleep disturbance has been associated with shift work, occupational stress, and musculoskeletal pain [35]. Transition to shift work can worsen sleep and mental-health outcomes [36], and shift-work disorder itself is common across shift-working populations [37]. Day-to-day evidence also suggests that sleep and pain influence one another over short timescales [38]. Within this sample, therefore, sleep variables did not explain multisite pain independently of the other included factors. Interventions that improve sleep can benefit people with chronic musculoskeletal pain in other settings [39], but intervention effects cannot be inferred from the present observational study.

### 4.1. Practical Implications and Future Research

In practical terms, these exploratory findings support four measures that occupational-health services for private security personnel could consider together: (i) periodic musculoskeletal surveillance that records the number of painful anatomical regions rather than a single index region, since single-region surveillance may underestimate the burden carried by officers with multisite symptoms; (ii) brief periodic screening for emotional exhaustion and other psychosocial demands; (iii) review of extended or variable schedules and of recovery opportunities; and (iv) ergonomic assessment of prolonged standing and walking, particularly for the low back, neck, and lower extremities. Randomized trials in other manual and industrial workforces indicate that structured ergonomic training combined with exercise can reduce non-specific low back pain and work-related musculoskeletal disorders [40,41], which suggests a feasible starting point for intervention studies in this group. Future research should be longitudinal and multicenter; incorporate objective ergonomic and organizational exposures (standing and walking time, postural load, shift type and sequence); measure related work-related mental-health constructs (occupational stress, fatigue, monotony, anxiety) alongside burnout to identify which best explains multisite pain; include female officers and other regions and sectors; and proceed to intervention trials combining ergonomic and psychosocial components. All such recommendations must be tested prospectively, because the present study did not evaluate any preventive or therapeutic intervention and its associations are exploratory.

### 4.2. Strengths and Limitations

Strengths of the study include the simultaneous assessment of musculoskeletal symptoms, burnout, sleep quality, and occupational characteristics in an understudied workforce; the use of established instruments; a high participation rate; reporting of the anatomical distribution of symptoms; analysis of pain as a multisite burden with count-based and ordinal sensitivity analyses rather than only as isolated regional complaints; a prespecified strategy for selecting the primary statistical test for each variable; and an extensive set of robustness analyses, including Firth penalization, alternative exposure specifications, and data-quality exclusions.

Several limitations require emphasis. First, the cross-sectional design precludes temporal or causal inference. Second, the recall periods of the main variables differ: musculoskeletal pain referred to the preceding 12 months, whereas the PSQI covers the previous month and the MBI reflects a current occupational state. The variables entered into the association analyses therefore do not represent the same temporal window, and current mood states may color the recollection of past somatic symptoms. Third, all participants were male and recruited from workplaces within a single district of one city, limiting generalizability to female officers, other regions, and other private-security roles; in addition, a healthy-worker survivor effect may lead to underestimation of the true symptom burden. Fourth, although the participation rate was high (86.6%), residual non-response bias cannot be fully excluded. Fifth, all measures were based on participants’ self-reports collected in face-to-face interviews and are therefore susceptible to recall and common-method bias, and interviewer administration may additionally introduce social-desirability bias; data quality control relied on range, consistency and plausibility checks rather than embedded validity items, and four records with implausible occupational values were retained in the primary analysis (results were unchanged when they were excluded). Sixth, objective ergonomic and organizational exposures—standing time, walking distance, postural load, primary working posture (static monitoring versus mobile patrolling), physical activity load, shift type and sequence, night-shift frequency, break duration, and footwear—were not measured, and the sector of each officer’s workplace was not recorded; their omission is a major limitation and precludes any comparison of psychosocial with biomechanical explanations. Seventh, none of the 99 exploratory region-specific comparisons remained significant after Benjamini–Hochberg correction, so all site-specific observations are hypothesis-generating only. Eighth, dichotomizing the number of painful regions into ≤2 versus ≥3 sites reduces information relative to modeling the site count directly; count-based and ordinal sensitivity analyses were therefore added, and the attenuation of the emotional-exhaustion association in these models indicates that the primary dichotomous estimate should be interpreted cautiously. Ninth, the logistic models included seven to ten explanatory variables for 52 events (approximately five to seven events per variable), the Nagelkerke R^2^ values were modest (approximately 0.20), and the primary model showed imperfect calibration, although calibration was adequate in the sensitivity and robustness models; residual confounding by unmeasured factors—including occupationally important variables such as detailed shift schedules and physical workload—cannot be excluded. Tenth, item-level scale data were not retained in the analysis dataset, so subscale-specific internal-consistency coefficients could not be computed, and the measurement properties of the MBI have not been formally established in private security personnel. Finally, no a priori sample-size calculation was performed; the width of the reported confidence intervals—for the primary emotional-exhaustion estimate, an odds increase of 2% to 23% per point—reflects the limited precision attainable at this sample size, and confirmation in a larger, prospectively designed, multicenter cohort is warranted.

## 5. Conclusions

In this exploratory study—to our knowledge the first to take the anatomical distribution and multisite burden of musculoskeletal pain as the primary outcome in private security officers—musculoskeletal pain was common: 82.7% reported symptoms in at least one anatomical region during the preceding 12 months and nearly half reported pain in three or more regions, with the greatest prevalence in the low back, neck, and ankles/feet. Among the burnout, sleep and occupational variables examined, emotional exhaustion showed the most consistent association with multisite pain; this association was positive in all model specifications but statistically significant only in some, and the exploratory, cross-sectional design precludes causal conclusions. In particular, no inference can be drawn about the relative importance of psychosocial versus biomechanical factors, because biomechanical and ergonomic exposures were not assessed. These results support considering musculoskeletal health in private security personnel within an integrated occupational framework that includes psychosocial as well as physical and organizational factors. Longitudinal, multicenter research incorporating objective ergonomic exposures, detailed shift characteristics, and repeated assessments is needed to clarify temporal pathways and inform prevention.

## Figures and Tables

**Table 1:** Sociodemographic and occupational characteristics of participants (*n* = 110).

Variable	Value
Sex, male, *n* (%)	110 (100.0)
Age, years, mean ± SD (range 21–56)	36.94 ± 9.02
21–29 years, *n* (%)	23 (20.9)
30–39 years, *n* (%)	42 (38.2)
40–49 years, *n* (%)	32 (29.1)
≥50 years, *n* (%)	13 (11.8)
Married, *n* (%)	62 (56.4)
Single, *n* (%)	48 (43.6)
Middle school education, *n* (%)	10 (9.1)
High school education, *n* (%)	71 (64.5)
University education, *n* (%)	23 (20.9)
Postgraduate education, *n* (%)	6 (5.5)
BMI, kg/m^2^, mean ± SD (range 19.29–33.95)	24.53 ± 3.26
Current smoker, *n* (%)	37 (33.6)
Non-smoker, *n* (%)	73 (66.4)
Years in private security, mean ± SD	11.02 ± 8.45
1–5 years, *n* (%)	39 (35.5)
6–10 years, *n* (%)	25 (22.7)
11–20 years, *n* (%)	31 (28.2)
>20 years, *n* (%)	15 (13.6)
Daily working hours, mean ± SD	10.64 ± 1.84
8–10 h/day, *n* (%)	40 (36.4)
12 h/day, *n* (%)	70 (63.6)
Weekly working hours, mean ± SD	49.40 ± 9.08
≤45 h/week, *n* (%)	56 (50.9)
46–54 h/week, *n* (%)	17 (15.5)
60 h/week, *n* (%)	37 (33.6)
Working days per month, mean ± SD	20.82 ± 5.52
≤19 days, *n* (%)	27 (24.5)
20–24 days, *n* (%)	49 (44.5)
≥25 days, *n* (%)	34 (30.9)

Data are presented as mean ± SD or *n* (%). Indented rows show the distribution of participants across categories of the corresponding variable. Percentages may not sum to 100 because of rounding. BMI, body mass index; SD, standard deviation.

**Table 2:** Twelve-month prevalence of musculoskeletal pain by anatomical region.

Anatomical Region	Pain Present, *n* (%)	95% CI	Pain Absent, *n* (%)
Low back	60 (54.5)	45.2–63.5	50 (45.5)
Neck	46 (41.8)	33.0–51.2	64 (58.2)
Ankles/feet	46 (41.8)	33.0–51.2	64 (58.2)
Upper back	35 (31.8)	23.9–41.0	75 (68.2)
Shoulders	32 (29.1)	21.4–38.2	78 (70.9)
Knees	31 (28.2)	20.6–37.2	79 (71.8)
Hips/thighs	22 (20.0)	13.6–28.4	88 (80.0)
Elbows	16 (14.5)	9.2–22.3	94 (85.5)
Wrists/hands	15 (13.6)	8.4–21.3	95 (86.4)

Pain refers to self-reported pain, ache, or discomfort during the preceding 12 months. Confidence intervals for prevalence were calculated using the Wilson score method. CI, confidence interval.

**Table 3:** Burnout, sleep, and occupational variables according to musculoskeletal pain burden.

Variable	Pain in ≤2 Regions (*n* = 58)	Pain in ≥3 Regions (*n* = 52)	Mean Difference (95% CI)	*p* (*t* Test)	*p* (Mann–Whitney U)	Cohen’s d
Emotional exhaustion	10.81 ± 6.77	14.13 ± 6.26	3.32 (0.86 to 5.78)	**0.009**	0.014	0.51
Depersonalization	5.34 ± 3.80	6.08 ± 3.25	0.73 (−0.60 to 2.06)	0.283	**0.202**	0.21
Personal accomplishment	13.97 ± 6.26	14.81 ± 5.30	0.84 (−1.34 to 3.02)	0.451	**0.376**	0.14
PSQI global score	3.21 ± 2.81	3.92 ± 2.90	0.72 (−0.36 to 1.80)	0.192	**0.159**	0.25
PSQI C1 subjective sleep quality	0.88 ± 0.68	1.10 ± 0.66	0.22 (−0.04 to 0.47)	0.094	**0.079**	0.32
PSQI C2 sleep latency	0.50 ± 0.73	0.54 ± 0.80	0.04 (−0.25 to 0.33)	0.793	**0.953**	0.05
PSQI C3 sleep duration	0.28 ± 0.45	0.31 ± 0.58	0.03 (−0.17 to 0.23)	0.747	**0.991**	0.06
PSQI C4 habitual sleep efficiency	0.33 ± 0.66	0.37 ± 0.69	0.04 (−0.22 to 0.29)	0.769	**0.731**	0.06
PSQI C5 sleep disturbances	0.69 ± 0.75	0.69 ± 0.67	0.00 (−0.27 to 0.27)	0.985	**0.804**	0.00
PSQI C6 use of sleep medication	0.14 ± 0.40	0.29 ± 0.50	0.15 (−0.02 to 0.32)	0.081	**0.053**	0.34
PSQI C7 daytime dysfunction	0.40 ± 0.79	0.63 ± 0.71	0.24 (−0.05 to 0.52)	0.103	**0.013**	0.31
Age, years	38.47 ± 8.92	35.23 ± 8.91	−3.23 (−6.61 to 0.14)	**0.060**	0.069	−0.36
Body mass index, kg/m^2^	25.15 ± 3.07	23.83 ± 3.35	−1.32 (−2.54 to −0.10)	0.033	**0.012**	−0.41
Years in private security	12.47 ± 8.44	9.40 ± 8.25	−3.06 (−6.22 to 0.09)	0.058	**0.026**	−0.37
Daily working hours	10.40 ± 1.89	10.90 ± 1.75	0.51 (−0.18 to 1.20)	0.149	**0.147**	0.28
Weekly working hours	47.50 ± 9.04	51.52 ± 8.74	4.02 (0.66 to 7.38)	0.020	**0.071**	0.45
Working days per month	20.50 ± 6.72	21.17 ± 3.79	0.67 (−1.36 to 2.71)	0.526	**0.670**	0.12

Data are mean ± SD. For each variable, the primary test (shown in bold) was designated a priori by measurement level and distribution: independent-samples *t* test where Shapiro–Wilk indicated approximate normality in both groups, and Mann–Whitney U test for ordinal PSQI component scores and variables departing from normality; the non-primary test is reported for transparency. Cohen’s d is the standardized effect size. Multisite pain was defined as pain in ≥3 anatomical regions during the preceding 12 months. CI, confidence interval; MBI, Maslach Burnout Inventory; PSQI, Pittsburgh Sleep Quality Index.

**Table 4:** Binary logistic regression analysis of factors associated with pain in ≥3 anatomical regions (*n* = 110; 52 events).

Variable	B	SE	Wald	OR	95% CI	*p* Value
Emotional exhaustion	0.112	0.048	5.54	1.12	1.02–1.23	0.019
Depersonalization	−0.094	0.080	1.38	0.91	0.78–1.06	0.241
Subjective sleep quality	0.637	0.376	2.87	1.89	0.90–3.95	0.090
Sleep-medication use	0.600	0.509	1.39	1.82	0.67–4.94	0.239
Daytime dysfunction	−0.443	0.380	1.36	0.64	0.30–1.35	0.244
Years in private security	−0.035	0.030	1.32	0.97	0.91–1.02	0.250
Weekly working hours	0.042	0.030	2.03	1.04	0.98–1.11	0.154

Outcome: pain in ≥3 anatomical regions (1) versus pain in ≤2 regions (0). Omnibus χ^2^(7) = 17.498, *p* = 0.014; Nagelkerke R^2^ = 0.196; Hosmer–Lemeshow χ^2^(8) = 17.494, *p* = 0.025; overall classification accuracy = 69.1%. B, regression coefficient; CI, confidence interval; OR, odds ratio; SE, standard error.

**Table 5:** Sensitivity analysis: binary logistic regression additionally adjusted for age and body mass index (*n* = 110; 52 events).

Variable	B	SE	Wald	OR	95% CI	*p* Value
Emotional exhaustion	0.108	0.048	5.04	1.11	1.01–1.22	0.025
Depersonalization	−0.088	0.081	1.18	0.92	0.78–1.07	0.277
Subjective sleep quality	0.694	0.385	3.25	2.00	0.94–4.26	0.072
Sleep-medication use	0.558	0.513	1.19	1.75	0.64–4.78	0.276
Daytime dysfunction	−0.466	0.391	1.42	0.63	0.29–1.35	0.234
Years in private security	−0.005	0.048	0.01	1.00	0.91–1.09	0.920
Weekly working hours	0.042	0.030	2.03	1.04	0.98–1.11	0.154
Age	−0.017	0.044	0.15	0.98	0.90–1.07	0.698
Body mass index	−0.074	0.077	0.92	0.93	0.80–1.08	0.337

Outcome: pain in ≥3 anatomical regions (1) versus pain in ≤2 regions (0). Omnibus χ^2^(9) = 18.720, *p* = 0.028; Nagelkerke R^2^ = 0.209; Hosmer–Lemeshow χ^2^(8) = 7.506, *p* = 0.483; overall classification accuracy = 65.5%. B, regression coefficient; CI, confidence interval; OR, odds ratio; SE, standard error.

## Data Availability

The datasets generated and/or analyzed during the current study are not publicly available due to participant privacy, confidentiality, and institutional ethical restrictions, as the data were collected at a small number of identifiable workplaces. De-identified data may be made available from the corresponding author upon reasonable request, subject to approval by the relevant institutional ethics committee and applicable data-sharing regulations.

## Supplementary Materials

The following supporting information can be downloaded at https://www.mdpi.com/article/10.3390/healthcare14172830/s1, Figure S1: Participant flow diagram; Table S1: STROBE Statement—checklist of items that should be included in reports of cross-sectional studies, with the location of each item in the manuscript; Table S2: All 99 exploratory region-specific comparisons (9 anatomical regions × 11 variables) of burnout, sleep and occupational characteristics according to 12-month pain in each region; Table S3: Number of painful anatomical regions and global PSQI score across sociodemographic and occupational subgroups (*n* = 110).
