# Supplementary material for: Multisite Musculoskeletal Pain and Associated Psychosocial and Occupational Factors in Male Private Security Officers: A Cross-Sectional Study
Source: Healthcare (Basel). 2026 Sep 3;14(17):2830. doi: 10.3390/healthcare14172830 (PMC13565971; doi:10.3390/healthcare14172830)
Supplement: Supplementary file 1 [file healthcare-14-02830-s001.zip › healthcare-4520387-supplementary.pdf]

Supplementary Materials

Multisite Musculoskeletal Pain and Associated Psychosocial and Occupational Factors in Male Private Security Officers: A Cross-Sectional Study

Ömer Şevgin, Büşra Kesgin, Saniye Koyuncu, Aysu Güngör and Suzan Aydın

Figure S1

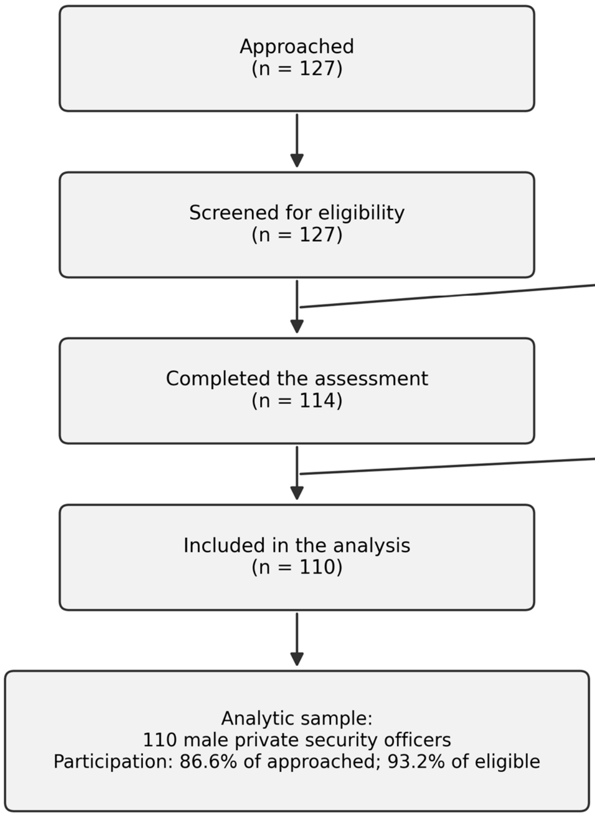

Figure S1. Participant flow diagram. Of the 127 private security officers approached, 9 did not meet the eligibility criteria and 4 declined to participate; of the 114 who completed the assessment, 4 were excluded because of incomplete questionnaires, yielding an analytic sample of 110 male private security officers (participation rate: 86.6% of those approached; 93.2% of those eligible).

Table S1. STROBE Statement — checklist of items that should be included in reports of cross-sectional studies, with the location of each item in the manuscript.

Item	No.	Recommendation	Location in manuscript
Title and abstract	1a	Indicate the study's design with a commonly used term in the title or the abstract	Title; Abstract
	1b	Provide in the abstract an informative and balanced summary of what was done and what was found	Abstract
Background/rationale	2	Explain the scientific background and rationale for the investigation being reported	Section 1
Objectives	3	State specific objectives, including any prespecified hypotheses	Section 1 (general objective; hypotheses H1–H3)
Study design	4	Present key elements of study design early in the paper	Section 2.1
Setting	5	Describe the setting, locations, and relevant dates, including periods of recruitment and data collection	Section 2.1
Participants	6	Give the eligibility criteria, and the sources and methods of selection of participants	Section 2.1
Variables	7	Clearly define all outcomes, exposures, predictors, potential confounders, and effect modifiers	Sections 2.2 and 2.3
Data sources/measurement	8	For each variable of interest, give sources of data and details of methods of assessment (measurement)	Sections 2.1 (interview procedure and interviewer training) and 2.2
Bias	9	Describe any efforts to address potential sources of bias	Sections 2.4 (data quality control, robustness analyses) and 4.2
Study size	10	Explain how the study size was arrived at	Section 2.1 (convenience sample; no a priori calculation)
Quantitative variables	11	Explain how quantitative variables were handled in the analyses; describe which groupings were chosen and why	Sections 2.3 and 2.4
Statistical methods	12a	Describe all statistical methods, including those used to control for confounding	Section 2.4
	12b	Describe any methods used to examine subgroups and interactions	Section 2.4; Tables S2 and S3
	12c	Explain how missing data were addressed	Section 2.1 (4 incomplete questionnaires excluded)
	12d	If applicable, describe analytical methods taking account of sampling strategy	Not applicable
	12e	Describe any sensitivity analyses	Sections 2.4 and 3.5
Participants (results)	13a	Report numbers of individuals at each stage of the study	Section 3.1; Figure S1
	13b	Give reasons for non-participation at each stage	Section 3.1; Figure S1
	13c	Consider use of a flow diagram	Figure S1
Descriptive data	14a	Give characteristics of study participants and information on exposures and potential confounders	Section 3.1; Table 1
	14b	Indicate number of participants with missing data for each variable of interest	Section 3.1 (complete-case analytic sample)
Outcome data	15	Report numbers of outcome events or summary measures	Section 3.2; Table 2
Main results	16a	Give unadjusted estimates and, if applicable,	Sections 3.3 and 3.4; Tables

		confounder-adjusted estimates and their precision; make clear which confounders were adjusted for and why	3–5
	16b	Report category boundaries when continuous variables were categorized	Sections 2.3, 2.4 and 3.5; Table 1
	16c	If relevant, consider translating estimates of relative risk into absolute risk	Not applicable
Other analyses	17	Report other analyses done — e.g., analyses of subgroups and interactions, and sensitivity analyses	Section 3.5; Tables S2 and S3
Key results	18	Summarise key results with reference to study objectives	Section 4, first paragraph (hypothesis outcomes)
Limitations	19	Discuss limitations of the study, taking into account sources of potential bias or imprecision	Section 4.2
Interpretation	20	Give a cautious overall interpretation of results considering objectives, limitations, multiplicity of analyses, and other relevant evidence	Sections 4 and 5
Generalisability	21	Discuss the generalisability (external validity) of the study results	Section 4.2
Funding	22	Give the source of funding and the role of the funders	Funding statement

Table S2. All 99 exploratory region-specific comparisons (9 anatomical regions × 11 variables) of burnout, sleep and occupational characteristics according to 12-month pain in each region.

Region	Variable	Pain absent (mean ± SD)	Pain present (mean ± SD)	p (t)	p (MW)	Primary	p (primary)	d	FDR-adj. p
Neck	Emotional exhaustion	11.75 ± 7.21	13.26 ± 5.93	0.246	0.235	t	0.246	0.225	0.645
Neck	Depersonalization	5.11 ± 3.26	6.50 ± 3.82	0.042	0.081	MW	0.081	0.397	0.532
Neck	Personal accomplishment	13.97 ± 6.01	14.91 ± 5.54	0.403	0.400	MW	0.400	0.162	0.734
Neck	PSQI global score	3.45 ± 2.95	3.67 ± 2.76	0.692	0.567	MW	0.567	0.077	0.861
Neck	PSQI C1 subjective sleep quality	0.95 ± 0.72	1.02 ± 0.61	0.602	0.578	MW	0.578	0.101	0.861
Neck	PSQI C6 use of sleep medication	0.22 ± 0.45	0.20 ± 0.45	0.793	0.722	MW	0.722	-0.051	0.894
Neck	PSQI C7 daytime dysfunction	0.41 ± 0.71	0.65 ± 0.82	0.096	0.069	MW	0.069	0.325	0.532
Neck	Age, years	38.11 ± 9.83	35.30 ± 7.55	0.108	0.140	t	0.108	-0.313	0.555
Neck	Body mass index	24.77 ± 3.37	24.19 ± 3.10	0.354	0.246	MW	0.246	-0.180	0.645
Neck	Years in private security	11.84 ± 8.81	9.87 ± 7.87	0.229	0.265	MW	0.265	-0.234	0.645
Neck	Weekly working hours	48.11 ± 9.43	51.20 ± 8.35	0.079	0.145	MW	0.145	0.343	0.599
Shoulders	Emotional exhaustion	11.49 ± 7.03	14.56 ± 5.36	0.028	0.015	t	0.028	0.466	0.407
Shoulders	Depersonalization	5.37 ± 3.68	6.47 ± 3.15	0.142	0.090	MW	0.090	0.310	0.532
Shoulders	Personal accomplishment	14.49 ± 6.08	14.06 ± 5.17	0.730	0.697	MW	0.697	-0.073	0.888
Shoulders	PSQI global score	3.26 ± 2.80	4.25 ± 2.93	0.098	0.066	MW	0.066	0.350	0.532
Shoulders	PSQI C1 subjective sleep quality	0.91 ± 0.67	1.16 ± 0.68	0.083	0.085	MW	0.085	0.367	0.532
Shoulders	PSQI C6 use of sleep medication	0.19 ± 0.46	0.25 ± 0.44	0.545	0.357	MW	0.357	0.127	0.727
Shoulders	PSQI C7 daytime dysfunction	0.49 ± 0.75	0.56 ± 0.80	0.640	0.563	MW	0.563	0.098	0.861
Shoulders	Age, years	36.78 ± 8.88	37.31 ± 9.49	0.781	0.841	t	0.781	0.059	0.927
Shoulders	Body mass index	24.82 ± 3.46	23.82 ± 2.62	0.144	0.217	MW	0.217	-0.309	0.645
Shoulders	Years in private security	10.99 ± 8.31	11.09 ± 8.92	0.952	0.971	MW	0.971	0.013	0.979
Shoulders	Weekly working hours	49.42 ± 9.29	49.34 ± 8.71	0.967	0.589	MW	0.589	-0.009	0.861
Upper back	Emotional exhaustion	12.71 ± 7.30	11.69 ± 5.27	0.460	0.660	t	0.460	-0.152	0.778
Upper back	Depersonalization	5.89 ± 3.65	5.26 ± 3.35	0.384	0.443	MW	0.443	-0.179	0.770
Upper back	Personal accomplishment	14.79 ± 5.93	13.46 ± 5.53	0.266	0.340	MW	0.340	-0.229	0.727
Upper back	PSQI global score	3.33 ± 2.85	4.00 ± 2.87	0.257	0.185	MW	0.185	0.233	0.645

Upper back	PSQI C1 subjective sleep quality	0.93 ± 0.64	1.09 ± 0.74	0.274	0.297	MW	0.297	0.225	0.684
Upper back	PSQI C6 use of sleep medication	0.19 ± 0.46	0.26 ± 0.44	0.448	0.267	MW	0.267	0.156	0.645
Upper back	PSQI C7 daytime dysfunction	0.48 ± 0.81	0.57 ± 0.65	0.561	0.200	MW	0.200	0.119	0.645
Upper back	Age, years	36.89 ± 9.24	37.03 ± 8.67	0.942	0.910	t	0.942	0.015	0.979
Upper back	Body mass index	24.73 ± 3.30	24.10 ± 3.19	0.353	0.401	MW	0.401	-0.191	0.734
Upper back	Years in private security	11.08 ± 8.44	10.89 ± 8.61	0.911	0.910	MW	0.910	-0.023	0.969
Upper back	Weekly working hours	49.31 ± 7.79	49.60 ± 11.51	0.876	0.648	MW	0.648	0.032	0.879
Elbows	Emotional exhaustion	12.05 ± 6.80	14.31 ± 6.03	0.215	0.306	t	0.215	0.337	0.645
Elbows	Depersonalization	5.62 ± 3.65	6.12 ± 2.96	0.600	0.485	MW	0.485	0.142	0.783
Elbows	Personal accomplishment	14.34 ± 5.79	14.50 ± 6.13	0.920	0.902	MW	0.902	0.027	0.969
Elbows	PSQI global score	3.62 ± 2.90	3.12 ± 2.66	0.528	0.498	MW	0.498	-0.171	0.783
Elbows	PSQI C1 subjective sleep quality	0.96 ± 0.65	1.12 ± 0.81	0.363	0.471	MW	0.471	0.247	0.778
Elbows	PSQI C6 use of sleep medication	0.19 ± 0.45	0.31 ± 0.48	0.324	0.207	MW	0.207	0.268	0.645
Elbows	PSQI C7 daytime dysfunction	0.54 ± 0.80	0.31 ± 0.48	0.267	0.399	MW	0.399	-0.302	0.734
Elbows	Age, years	36.77 ± 8.98	37.94 ± 9.46	0.633	0.575	t	0.633	0.129	0.879
Elbows	Body mass index	24.57 ± 3.24	24.26 ± 3.48	0.724	0.700	MW	0.700	-0.096	0.888
Elbows	Years in private security	10.83 ± 8.27	12.12 ± 9.67	0.573	0.715	MW	0.715	0.153	0.894
Elbows	Weekly working hours	49.78 ± 9.07	47.19 ± 9.12	0.294	0.084	MW	0.084	-0.285	0.532
Wrists/hands	Emotional exhaustion	12.01 ± 6.74	14.73 ± 6.24	0.145	0.194	t	0.145	0.408	0.599
Wrists/hands	Depersonalization	5.53 ± 3.61	6.73 ± 3.13	0.223	0.159	MW	0.159	0.340	0.605
Wrists/hands	Personal accomplishment	14.41 ± 5.81	14.07 ± 6.03	0.832	0.831	MW	0.831	-0.059	0.956
Wrists/hands	PSQI global score	3.53 ± 2.82	3.67 ± 3.24	0.861	0.979	MW	0.979	0.049	0.979
Wrists/hands	PSQI C1 subjective sleep quality	0.94 ± 0.65	1.27 ± 0.80	0.079	0.113	MW	0.113	0.492	0.555
Wrists/hands	PSQI C6 use of sleep medication	0.18 ± 0.41	0.40 ± 0.63	0.078	0.118	MW	0.118	0.495	0.555
Wrists/hands	PSQI C7 daytime dysfunction	0.53 ± 0.78	0.40 ± 0.63	0.554	0.645	MW	0.645	-0.165	0.879
Wrists/hands	Age, years	36.93 ± 8.75	37.00 ± 10.93	0.977	1.000	t	0.977	0.008	0.979
Wrists/hands	Body mass index	24.65 ± 3.30	23.75 ± 2.97	0.326	0.420	MW	0.420	-0.274	0.757
Wrists/hands	Years in private security	11.04 ± 8.29	10.87 ± 9.71	0.941	0.757	MW	0.757	-0.021	0.925
Wrists/hands	Weekly working hours	49.57 ± 8.99	48.33 ± 9.94	0.627	0.236	MW	0.236	-0.135	0.645
Low back	Emotional	11.48 ±	13.13 ±	0.200	0.229	t	0.200	0.247	0.645

	exhaustion	7.04	6.39						
Low back	Depersonalization	5.46 ± 3.94	5.88 ± 3.22	0.536	0.394	MW	0.394	0.119	0.734
Low back	Personal accomplishment	14.12 ± 6.28	14.57 ± 5.44	0.690	0.678	MW	0.678	0.077	0.883
Low back	PSQI global score	3.00 ± 2.71	4.00 ± 2.93	0.068	0.048	MW	0.048	0.353	0.523
Low back	PSQI C1 subjective sleep quality	0.90 ± 0.65	1.05 ± 0.70	0.249	0.224	MW	0.224	0.222	0.645
Low back	PSQI C6 use of sleep medication	0.20 ± 0.45	0.22 ± 0.45	0.848	0.805	MW	0.805	0.037	0.938
Low back	PSQI C7 daytime dysfunction	0.40 ± 0.76	0.60 ± 0.76	0.172	0.066	MW	0.066	0.263	0.532
Low back	Age, years	37.06 ± 9.09	36.83 ± 9.03	0.896	0.904	t	0.896	-0.025	0.969
Low back	Body mass index	24.80 ± 3.36	24.30 ± 3.19	0.424	0.397	MW	0.397	-0.154	0.734
Low back	Years in private security	11.14 ± 8.40	10.92 ± 8.57	0.891	0.787	MW	0.787	-0.026	0.927
Low back	Weekly working hours	48.52 ± 7.62	50.13 ± 10.15	0.356	0.253	MW	0.253	0.177	0.645
Hips/thighs	Emotional exhaustion	11.91 ± 6.64	14.27 ± 6.83	0.140	0.188	t	0.140	0.354	0.599
Hips/thighs	Depersonalization	5.47 ± 3.62	6.59 ± 3.22	0.186	0.156	MW	0.156	0.318	0.605
Hips/thighs	Personal accomplishment	14.25 ± 5.55	14.82 ± 6.89	0.684	0.611	MW	0.611	0.097	0.864
Hips/thighs	PSQI global score	3.60 ± 2.79	3.32 ± 3.18	0.679	0.358	MW	0.358	-0.099	0.727
Hips/thighs	PSQI C1 subjective sleep quality	0.95 ± 0.64	1.09 ± 0.81	0.401	0.493	MW	0.493	0.201	0.783
Hips/thighs	PSQI C6 use of sleep medication	0.19 ± 0.43	0.27 ± 0.55	0.462	0.591	MW	0.591	0.176	0.861
Hips/thighs	PSQI C7 daytime dysfunction	0.51 ± 0.76	0.50 ± 0.80	0.951	0.872	MW	0.872	-0.015	0.969
Hips/thighs	Age, years	37.00 ± 8.92	36.68 ± 9.64	0.883	0.828	t	0.883	-0.035	0.969
Hips/thighs	Body mass index	24.47 ± 3.22	24.74 ± 3.49	0.736	0.896	MW	0.896	0.080	0.969
Hips/thighs	Years in private security	10.97 ± 8.16	11.23 ± 9.75	0.897	0.857	MW	0.857	0.031	0.969
Hips/thighs	Weekly working hours	49.68 ± 9.16	48.27 ± 8.88	0.518	0.218	MW	0.218	-0.155	0.645
Knees	Emotional exhaustion	12.01 ± 7.14	13.32 ± 5.49	0.360	0.423	t	0.360	0.195	0.727
Knees	Depersonalization	5.48 ± 3.67	6.23 ± 3.25	0.325	0.271	MW	0.271	0.209	0.645
Knees	Personal accomplishment	14.73 ± 5.98	13.42 ± 5.33	0.288	0.313	MW	0.313	-0.226	0.689
Knees	PSQI global score	3.46 ± 2.82	3.77 ± 3.00	0.602	0.610	MW	0.610	0.111	0.864
Knees	PSQI C1 subjective sleep quality	0.97 ± 0.66	1.00 ± 0.73	0.861	0.949	MW	0.949	0.037	0.979
Knees	PSQI C6 use of sleep medication	0.15 ± 0.40	0.35 ± 0.55	0.033	0.029	MW	0.029	0.457	0.407
Knees	PSQI C7 daytime dysfunction	0.52 ± 0.81	0.48 ± 0.63	0.829	0.778	MW	0.778	-0.046	0.927
Knees	Age, years	37.16 ± 9.16	36.35 ± 8.77	0.674	0.772	t	0.674	-0.089	0.883

Knees	Body mass index	24.51 ± 3.16	24.57 ± 3.56	0.937	0.966	MW	0.966	0.017	0.979
Knees	Years in private security	11.46 ± 8.60	9.90 ± 8.08	0.389	0.468	MW	0.468	-0.183	0.778
Knees	Weekly working hours	48.97 ± 9.24	50.48 ± 8.74	0.436	0.661	MW	0.661	0.166	0.883
Ankles/feet	Emotional exhaustion	11.08 ± 6.94	14.20 ± 5.99	0.016	0.028	t	0.016	0.475	0.349
Ankles/feet	Depersonalization	5.30 ± 3.77	6.24 ± 3.18	0.171	0.113	MW	0.113	0.266	0.555
Ankles/feet	Personal accomplishment	13.94 ± 5.97	14.96 ± 5.60	0.367	0.308	MW	0.308	0.175	0.689
Ankles/feet	PSQI global score	3.22 ± 2.84	4.00 ± 2.86	0.159	0.091	MW	0.091	0.274	0.532
Ankles/feet	PSQI C1 subjective sleep quality	0.92 ± 0.65	1.07 ± 0.71	0.275	0.273	MW	0.273	0.212	0.645
Ankles/feet	PSQI C6 use of sleep medication	0.14 ± 0.39	0.30 ± 0.51	0.060	0.042	MW	0.042	0.367	0.518
Ankles/feet	PSQI C7 daytime dysfunction	0.39 ± 0.75	0.67 ± 0.76	0.054	0.013	MW	0.013	0.376	0.349
Ankles/feet	Age, years	38.66 ± 8.63	34.54 ± 9.09	0.018	0.012	t	0.018	-0.466	0.349
Ankles/feet	Body mass index	25.26 ± 3.09	23.51 ± 3.25	0.005	0.001	MW	0.001	-0.553	0.082
Ankles/feet	Years in private security	12.91 ± 8.22	8.39 ± 8.14	0.005	0.002	MW	0.002	-0.551	0.082
Ankles/feet	Weekly working hours	48.39 ± 9.15	50.80 ± 8.90	0.170	0.437	MW	0.437	0.267	0.770

For each variable, the primary test (t = independent-samples t test; MW = Mann–Whitney U test) was designated a priori by measurement level and distribution (Section 2.4). FDR-adj. p, Benjamini–Hochberg false-discovery-rate-adjusted p value computed across all 99 primary p values; no comparison remained significant after adjustment (lowest adjusted p = 0.082). d, Cohen's d; MW, Mann–Whitney U; SD, standard deviation.

Table S3. Number of painful anatomical regions and global PSQI score across sociodemographic and occupational subgroups (n = 110).

Grouping variable	Outcome	Groups (mean ± SD)	p	FDR-adj. p
Marital status	Number of painful regions	Single: 2.52±1.87 vs Married: 2.94±2.67	0.779	0.915
Marital status	PSQI global	Single: 3.48±2.83 vs Married: 3.60±2.91	0.873	0.915
Smoking	Number of painful regions	Smoker: 3.05±2.62 vs Non-smoker: 2.60±2.20	0.466	0.915
Smoking	PSQI global	Smoker: 3.62±2.95 vs Non-smoker: 3.51±2.84	0.915	0.915
Education (4 groups)	Number of painful regions	1.70 ∣ 2.87 ∣ 3.22 ∣ 1.33	0.164	0.915
Education (4 groups)	PSQI global	4.30 ∣ 3.49 ∣ 3.09 ∣ 4.67	0.829	0.915

Two-group comparisons used the Mann–Whitney U test; the four-group education comparison used the Kruskal–Wallis test (group means shown in the order middle school ∣ high school ∣ university ∣ postgraduate). p values were adjusted with the Benjamini–Hochberg procedure; no comparison was statistically significant. PSQI, Pittsburgh Sleep Quality Index; SD, standard deviation.
